# No Right Way: Removal of a Pencil Foreign Body From a Male Urethra

**DOI:** 10.7759/cureus.74860

**Published:** 2024-11-30

**Authors:** Fraser Barbour, John Hayes, Ahmad Khalifa

**Affiliations:** 1 Urology, Sunderland Royal Hospital, Sunderland, GBR

**Keywords:** dangerous sexual practices, emergency urology, foreign bodies, foreign body retrieval, urology trauma

## Abstract

Foreign body insertion into the urethra is uncommonly encountered in urology practice. Such insertion can result in a myriad of problems including bleeding, infectious sequelae, urinary retention, urethral injury or rupture, and resultant urethral stricture formation. This article describes a case in which an elderly male inserted a pencil into his urethra, which subsequently became lodged and required removal under regional anesthesia in the operating theater. The foreign body resulted in urethral damage in the form of a false passage within the bulbomembranous urethra. The pencil was removed with forceps, followed by undertaking a check rigid ureteroscopy and cystoscopy before placement of a urethral catheter via a guidewire. Postoperatively, the patient developed urinary sepsis requiring an extended course of intravenous antibiotics. The catheter was left in situ for six weeks before removal to allow satisfactory urethral healing.

## Introduction

The insertion of foreign bodies into the urethra is uncommonly encountered in emergency medicine or urological practice [1]. The scientific term for the insertion of foreign bodies into the body is polyembolokoilamania [2]. Documented reasons for foreign body insertion down the urethra include sexual stimulation, mental health illness, cognitive impairment, self-injurious behavior, and drug or alcohol intoxication [3]. A vast array of objects have been reported in the literature as urethral foreign bodies [4,5]. Foreign bodies inserted into the urethra can remain there or pass into the bladder [6]. Patients who insert foreign bodies into the urethra often have a history of previous foreign body insertions or the presence of more than one foreign body when they present to the hospital [7].

The insertion of foreign bodies into the urethra is more common in men than in women, with one case series from a Chicago institution (2016) reporting that 26 of the 27 patients admitted to their hospital with urethral foreign bodies were male [8]. Imaging can be used to help confirm the presence of foreign bodies and determine both the number and position [9]. X-rays are often used initially; however, they can only detect radio-opaque foreign bodies [10]. Therefore, ultrasound, CT, or direct visualization with cystoscopy are sometimes required.

In this report, we present a case in which a pencil was inserted into a male urethra, which required surgical removal in the operating theater.

## Case presentation

An 83-year-old gentleman presented to our emergency department after self-inserting a pencil into his urethra. The reason given for inserting the foreign object was sexual pleasure. He had no past medical history of mental health issues or cognitive impairment. After insertion, the pencil could not be removed, and he developed difficulty with voiding, only able to pass small volumes. He presented to the hospital several days later after his penis became more swollen and painful.

He had several comorbidities, including ischemic heart disease, previous coronary artery bypass grafting, cardiac pacemaker, hypertension, type 2 diabetes mellitus, emphysema, and a previous pulmonary embolism. He was taking aspirin but was not on any anticoagulants. He had poor mobility and significant dyspnea with minimal exertion.

**Figure 1:**
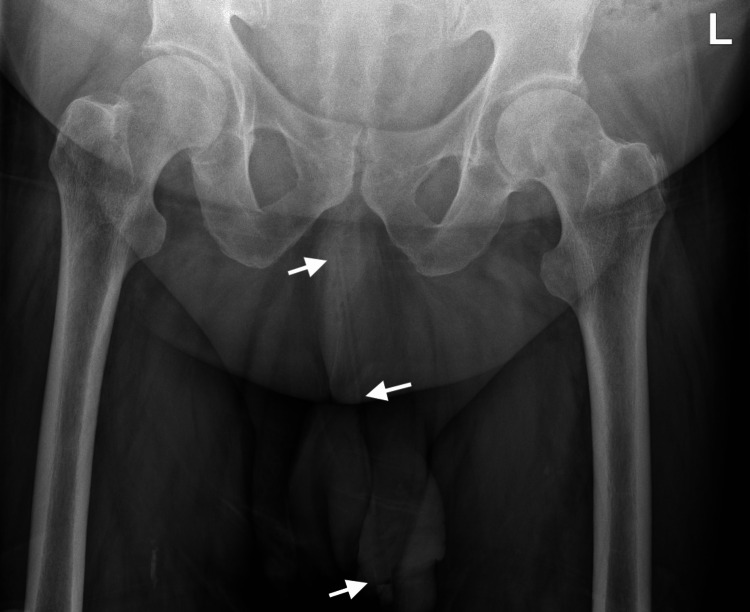
Pelvic x-ray showing a radiopaque pencil extending along the urethral tract, with the tip located proximally near the penile base

His observations were normal. Blood tests demonstrated an elevated white cell count of 14.9 and a CRP of 165. On examination, his penile shaft was evidently swollen and erythematous, with foul-smelling discharge from his urethral meatus. A long, thin, and firm foreign body, consistent with a pencil, was palpable within the entirety of his penile urethra and down to the penile base. The distal end was palpable approximately a centimeter from his urethral meatus. A manual attempt at removal was made, with Instillagel lubricant and local anesthetic; however, this was poorly tolerated by the patient. A plain radiograph was obtained, which visualized the urethral foreign body with the pencil tip located most proximally (Figure 1).

The patient was promptly started on empirical intravenous antibiotics and consented to foreign body extraction in the theater using rigid cystoscopy. He was reviewed by the on-call anesthetic team and, due to his comorbidities, was deemed a high-risk candidate for general anesthesia. So, he was given a regional block in the form of spinal anesthesia.

**Figure 2:**
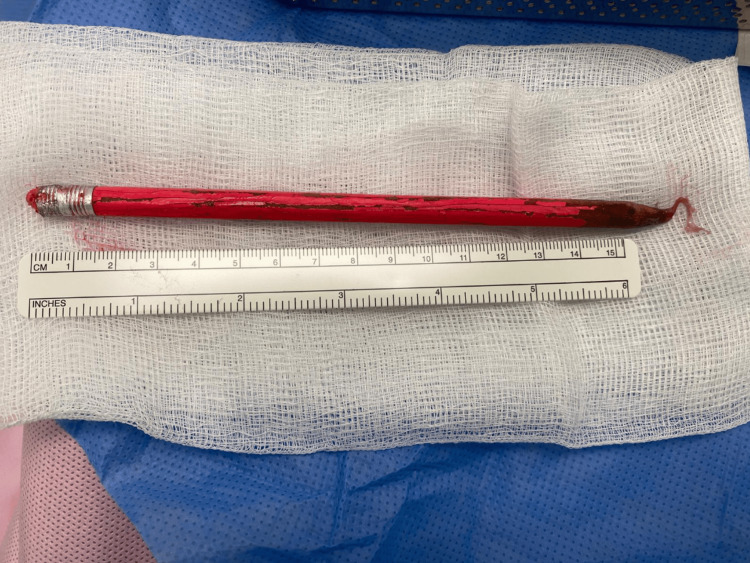
Photo showing the pencil after removal from the urethra

In the operating theater, after spinal anesthesia, he was placed in the lithotomy position. Additional Instillagel lubricant was used, and attempts were made to remove the pencil using a range of forceps and surgical clips. The pencil was successfully removed intact (Figure 2) using a pair of straight mosquito artery forceps. Rigid cystoscopy was then performed, which identified a false passage and urethral injury on the right bulbomembranous urethra. The external urethral sphincter and prostatic urethra appeared undamaged, and the lateral prostatic lobes were moderately occlusive. The bladder contained a large volume of foul-smelling urine, which was drained via the cystoscope, but there were no areas of injury within the bladder. Given the false passage and urethral injury, a guidewire was inserted into the bladder through the cystoscope before an open-tipped urethral catheter was railroaded over this into a satisfactory position.

Postoperatively, the patient developed pyrexia, tachycardia, hypotension, and a new oxygen requirement, consistent with evolving urinary sepsis. He required a prolonged inpatient stay and several days of IV antibiotics. The postoperative plan included catheter removal in six weeks, with outpatient follow-up in the clinic. He has been considered at significant risk of developing urethral strictures.

## Discussion

This case highlights the dangers of foreign body insertion, with our patient developing associated sepsis and potentially chronic problems with urethral stricture disease.

Urethral foreign bodies can be challenging to manage, and care should be taken to ensure that neither the object nor the instruments used cause further damage during removal [11]. Multiple factors may influence the decision-making regarding the removal technique, such as the foreign body material, whether it is blunt or sharp, the number of foreign bodies, and their location within the lower urinary tract.

Bezinque et al. [4] described a case in which a patient, who had presented multiple times with urethral foreign bodies, was managed conservatively for a small, blunt foreign body in the distal urethra. He was monitored with serial bladder scans until he was able to void, expelling the foreign body. Non-operative measures for the removal of urethral foreign bodies include milking, forceps removal if the object is visible at the end of the external urethral meatus [11], or as described by Crawford et al. [12], placing a pediatric catheter past the foreign body under ultrasound guidance, then inflating the catheter balloon to 2 ml before removing the catheter and trying to bring the foreign object out alongside it. If these measures fail, subsequent steps that could be considered include cystoscopic removal using stent graspers, stone baskets, or biopsy forceps [4]. Further techniques include open surgical removal, which, depending on the location of the foreign body, could involve meatotomy, external urethrotomy, or suprapubic cystotomy [11].

Once the foreign body has been removed and if there is significant associated urethral injury, a urethral catheter may need to be inserted, preferably under cystoscopic guidance over a guidewire, while the urethra is given time to heal. In severe injuries, a suprapubic catheter may be the only possible form of bladder drainage [13].

There is often a delay in seeking medical consultation in patients with urethral foreign bodies due to embarrassment [11,14]. This can lead to more significant damage to the urethra or other structures and increase the risk of associated urinary infections. Some sources recommend routine psychiatry referral in all cases of foreign body insertion to investigate potential underlying psychiatric illnesses [1,14,15], whereas other studies note that this is not a standard practice everywhere, and referral may be decided on a case-by-case basis, or in patients with recurrent episodes [7,11].

## Conclusions

There are various reasons why patients may insert urethral foreign bodies. This case highlights how insertion can lead to significant trauma to the urogenital tract and may be associated with life-threatening sequelae, such as urinary sepsis. Multiple methods are available for managing urethral foreign bodies. The focus should be on prompt removal while minimizing further injury to the urinary tract. The underlying reasons for urethral foreign body insertion should be explored, and a psychiatry referral should be considered when appropriate.
